# Feasibility of blinding spinal manual therapy interventions among participants and outcome assessors: protocol for a blinding feasibility trial

**DOI:** 10.1186/s40814-024-01492-6

**Published:** 2024-05-02

**Authors:** Javier Muñoz Laguna, Astrid Kurmann, Léonie Hofstetter, Emanuela Nyantakyi, Lauren Clack, Heejung Bang, Nadine E. Foster, Julia Braun, Milo A. Puhan, Mazda Farshad, Cesar A. Hincapié

**Affiliations:** 1 EBPI-UWZH Musculoskeletal Epidemiology Research Group, University of Zurich and Balgrist University Hospital, Zurich, Switzerland; 2 Epidemiology, Biostatistics and Prevention Institute (EBPI), University of Zurich, Zurich, Switzerland; 3 University Spine Centre Zurich (UWZH), Balgrist University Hospital, University of Zurich, Zurich, Switzerland; 4 Institute for Implementation Science in Health Care (IfIS), Medical Faculty, University of Zurich, Zurich, Switzerland; 5 Department of Infectious Diseases and Hospital Epidemiology, University Hospital Zurich, Zurich, Switzerland; 6 Division of Biostatistics, Department of Public Health Sciences, School of Medicine, University of California, Davis, United States; 7 Surgical Treatment and Rehabilitation Service (STARS), STARS Education and Research Alliance, The University of Queensland and Metro North Health, Brisbane, Australia; 8 School of Medicine, Keele University, Keele, United Kingdom; 9 Department of Orthopedics, Balgrist University Hospital, University of Zurich, Zurich, Switzerland

**Keywords:** Trial protocol, Spinal manipulation, Low back pain, Double-blind method, Trial methods, Blinding

## Abstract

**Introduction:**

Blinding is a methodologically important aspect in randomised controlled trials yet frequently overlooked in trials of spinal manual therapy interventions for back pain. To help inform the blinding methods of a future, double-placebo-controlled trial comparing spinal manual therapy and nerve root injection for lumbosacral radicular pain, we set four objectives: (1) to assess the feasibility of blinding participants, randomly allocated to an active or placebo-control spinal manual therapy intervention protocol, (2) to assess the feasibility of blinding outcome assessors within the trial, (3) to explore the influence of spinal manual therapy experience and low back pain on blinding, and (4) to explore factors contributing to perceptions about intervention assignment among participants and outcome assessors.

**Methods and analysis:**

Two-parallel-group, single-centre, placebo-controlled, methodological blinding feasibility randomised trial. We will recruit between 60 and 100 adults with or without back pain and with or without experience of spinal manual therapy from Zurich, Switzerland. Participants will be randomised to either an active spinal manual therapy or a placebo-control spinal manual therapy protocol—both interventions delivered over two study visits, up to two weeks apart. The primary outcome is participant blinding using the Bang blinding index within each intervention arm immediately after each of the two study visits. Secondary outcomes are participant blinding using the James blinding index, outcome assessor blinding (Bang and James blinding indices), self-reported factors influencing perceived intervention assignment among participants and outcome assessors, and participant-reported credibility and expectancy of study interventions. Other outcomes—included to blind the study objective from participants—are lumbar spine range of motion, self-rated general health, satisfaction with care, pain intensity, and function. Intervention provider outcomes include intervention component fidelity and quality of intervention delivery.

**Ethics and dissemination:**

The independent ethics commission of Canton Zurich granted ethical approval for this study (KEK 2023–00381). Written informed consent will be obtained from all participants. Findings will be disseminated in scientific conferences and a peer-reviewed publication and inform the blinding methods of a future double-placebo controlled trial comparing spinal manual therapy and nerve root injection for lumbosacral radicular pain—the SALuBRITY trial.

**Trial registration:**

NCT05778396.

**Supplementary Information:**

The online version contains supplementary material available at 10.1186/s40814-024-01492-6.

## Introduction

Blinding—also called masking [1]—of interventions in randomised controlled trials (RCTs) mitigates biases that can threaten the internal validity of estimated treatment effects [2]. By withholding intervention assignment information from participants and outcome assessors, performance and detection biases can be minimised [3]. Successful blinding of participants may also foster retention and compliance with intervention protocols. To assess the success of blinding, participants, outcome assessors, and persons in other trial roles (i.e. intervention providers, data analysts, investigators) can be asked about their beliefs about intervention assignment. Blinding of participants throughout the study period can be an ambitious undertaking in RCTs of physical interventions, since participants may correctly identify (i.e., generate correct beliefs about) their treatment assignment [4]. In RCTs of spinal manual therapy (SMT), relatively little is known about the blinding status of participants and outcome assessors [5, 6].

Designing placebo-control interventions for SMT interventions, as well as other physical interventions, is inherently challenging [7]. Nevertheless, some attempts at placebo- or sham-control intervention design and assessment of blinding in RCTs of SMT have been made [8–12], with varying study populations, blinding assessment methods, and measurement timepoints. Studies that have asked about beliefs about intervention assignment and attempted to assess and report blinding success have not consistently relied on proposed blinding assessment statistical methods [13], which limit their interpretability and comparability.

Lack of information on blinding status of participants and outcome assessors and lack of formal blinding assessments in RCTs of SMT create a methodological gap, as suboptimal blinding may lead to biased treatment effect estimates [14], compromising the quality and interpretation of the evidence [15]. The quantitative assessment of blinding among participants and outcome assessors—although often overlooked—is an important and worthwhile methodological endeavour in RCTs of SMT interventions.

### Objectives

To inform the blinding methods of a future, double-placebo-controlled RCT comparing SMT and corticosteroid nerve root injection for the management of patients with lumbosacral radicular pain (i.e., spine-related leg pain)—the SALuBRITY randomised clinical trial (ISRCTN87156139)—we prespecify four objectives. First, we will assess the feasibility of blinding participants, with or without experience of SMT or current back pain, randomly allocated to either active or placebo-control SMT intervention protocols. Second, we will assess the feasibility of blinding outcome assessors—clinicians or clinicians in training that interact with participants and are responsible for assessing pre- and post-intervention session range of motion outcomes—within the RCT setting. Third, we will explore the influence of SMT experience in the past three months and the presence of low back pain during the past four weeks (3 or greater on a 0 to 10 numeric rating scale [NRS]) on participant and outcome assessor blinding. Fourth, we will explore factors contributing to participant and outcome assessor beliefs about assigned intervention using a qualitative thematic analysis.

## Methods

### Trial design and registration

**Fig. 1:**
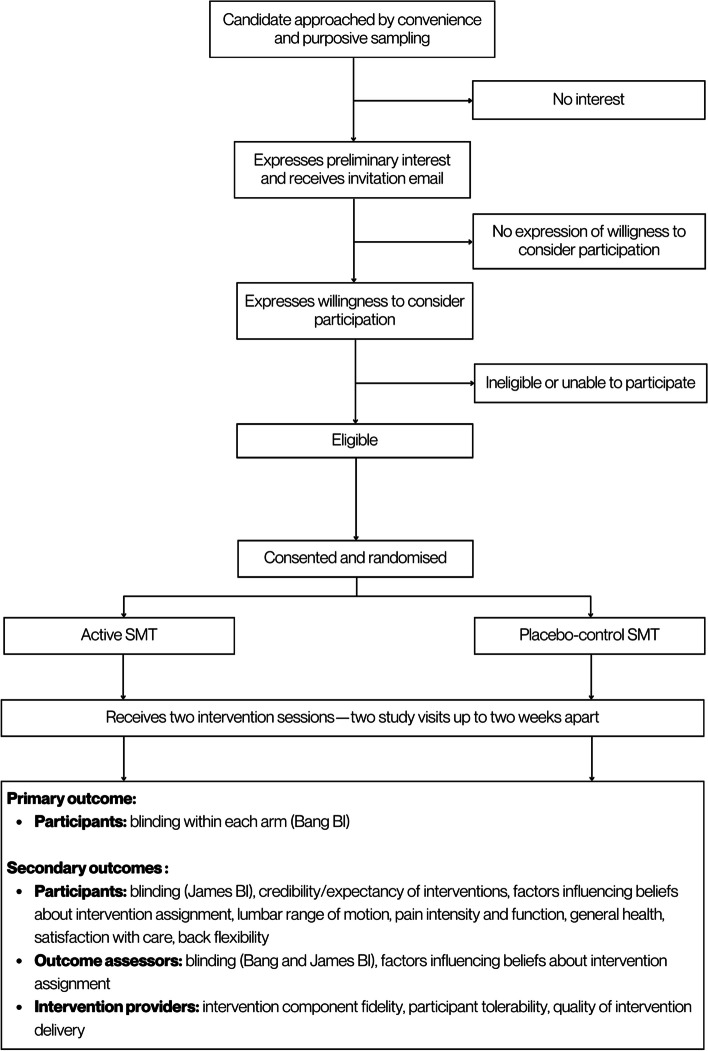
Trial design

Two-parallel-group (allocation ratio 1:1), single-centre, placebo-controlled, randomised blinding feasibility trial [16]. This trial protocol is reported in accordance with the SPIRIT 2013 guidance for protocols of clinical trials (Additional file 1) [17]. Our trial was preregistered on ClinicalTrials.gov: NCT05778396. Figure 1 details the trial design.

### Trial setting

Data will be collected, and interventions delivered at a community-based chiropractic research clinic in Zurich, Switzerland—CHIROMED Praxis im Seefeld (Dufourstrasse 101, 8008 Zurich). This research clinic actively collaborates and is affiliated with the EBPI-UWZH Musculoskeletal Epidemiology Research Group, within the Epidemiology, Biostatistics and Prevention Institute of the University of Zurich and the University Spine Centre Zurich of Balgrist University Hospital.

### Participants

#### Eligibility criteria

We will include participants aged 18 years or older with or without experience of SMT or current low back pain, to maximise recruitment feasibility of this study. Candidate participants—‘candidates’ hereafter—will be excluded if they have serious spinal pathology (e.g., spinal fracture, cancer or infection) or a history of lumbar spine surgery; are currently under care or in consultation with a specialist, chiropractor, physiotherapist, or osteopath for current low back pain; are a manual medicine health care provider (i.e., chiropractor, physiotherapist, osteopath, massage therapist, manual medicine trained physician); have a serious comorbidity preventing them from attending the research clinic and receiving the interventions; are pregnant or breastfeeding; are involved in pending litigation related to back pain; or are already participating in another research study related to back pain.

### Recruitment, screening, and informed consent

Candidates will be recruited by convenience and purposive sampling via word of mouth, snowballing, and mass email advertisements through the University of Zurich and Balgrist University Hospital mailing lists. Candidates will be approached for interest directly by study team members in and around the Zurich area. Candidates that express preliminary interest will receive an invitation email with study details. Those expressing willingness to consider participation will be able to prebook their study visit appointments and will receive a REDCap study eligibility survey [18]. Eligible candidates will be directed to review the study participant information form and complete an electronic signature in REDCap to express interest and willingness to participate. Participants will be blinded to the study objectives as the study information and consent form state that the aim of the study is to find out whether two SMT interventions are ‘practical and acceptable’ for a future study in patients with back pain (Additional file 2). Active SMT is described in the study information and consent form as an ‘active or real treatment, with an unknown exact benefit’. Placebo-control SMT is described as ‘other manual therapy procedure that is a comparison or control treatment that is not known to have a benefit’.

At the first clinic appointment, the trial coordinator reviews the participant information form with the candidate, answers any questions, confirms candidate understanding and agreement to participate, and requests the candidate to sign the official study informed consent form. Candidates completing these steps are deemed enrolled study participants.

### Interventions

SMT is conceptualised as a pragmatic hands-on treatment directed towards the spine that includes manipulation, mobilisation, or both [15]. Spinal manipulation is manual therapy applied to the spine that involves a high velocity, low amplitude impulse, or thrust applied at or near the end of the passive range of motion of the spinal joints [19]. Spinal mobilisation is manual therapy involving passive movement applied to a spinal region or segment that incorporates movements within spinal passive range of motion with the aim of achieving a therapeutic effect [20]. Mobilisation includes spinal traction, which is application of an intermittent or continuous distractive force within the passive range of motion.

Up to 10 licensed Doctors of Chiropractic Medicine intervention providers (‘intervention providers’ hereafter) with at least three years of clinical experience will be trained in the delivery of active and placebo-control SMT interventions according to prespecified intervention descriptions and standard operating procedures (see Additional file 3A) for consistent study interactions with participants and high-fidelity intervention delivery.

Up to eight outcome assessors—clinicians or clinicians in training not delivering the interventions—will be trained in range of motion outcome assessment procedures. Outcome assessors will collect ROM measurements immediately before and after intervention sessions. During training, outcome assessors will gain competence in standardised ROM data collection and interactions with participants within the RCT setting (see Additional file 3A for details). Importantly, outcome assessors will be blinded to the complete study protocol and objectives and will be told that the aim of the study is to assess the feasibility of two SMT interventions and improve the quality of a future study.

Participants will receive two sessions of the assigned intervention, up to two weeks apart, to maximise retention and minimise the risk for missing data. Participants who are lost to follow-up after study visit 1 will be asked for reasons for discontinuation. Due to our blinding feasibility primary objective, concomitant care is not restricted outside of the trial.

### Active SMT protocol

Active SMT includes three procedures: (1) side-lying lumbar spine manipulation, (2) prone lumbar spine mobilisation, and (3) prone thoracic spine manipulation—all delivered with therapeutic intent. These components are chosen based on their acceptability in clinical practice and their validated placebo-control counterparts [21]. The intervention provider will deliver (1) a high-velocity, low-amplitude (HVLA) thrust to the L4–L5 or L5–S1 spinal motion segments. Side-lying lumbar spine manipulation is performed bilaterally, with the intervention provider choosing any suitable technique and with or without occurrence of the characteristic joint cavitation associated with spine manipulation. The intervention provider then delivers (2) prone lumbar mobilisation by placing the contact hand and applying downward pressure over L4–L5 or L5–S1 with the other hand guiding a manual flexion-distraction piece through three sets of five full-range oscillations. The intervention provider then delivers (3) prone thoracic manipulation by applying a HVLA thrust with therapeutic intent in a posterior-to-anterior direction at the T5–T6 or T6–T7 spinal motion segments. The proposed ‘active’ element for procedures (1) and (3) is the high-velocity, low-amplitude thrust through the target spinal motion segments (i.e. L4–L5/L5–S1 in [1] and T5–T6/T6–T7 in [3]), potentially leading to neurophysiological effects [20]. For procedure (2), the proposed ‘active’ elements are the pressure, flexion, and distraction components through the target spinal motion segments (i.e. L4–L5/L5–S1). Details on the active SMT protocol—consistent with the 12-item TIDieR checklist [22]—are found in Additional file 3B.

### Placebo-control SMT protocol

Placebo-control SMT involves three procedures: (1) control side-lying lumbar spine manipulation, (2) control prone lumbar spine mobilisation, and (3) control prone thoracic spine manipulation—all performed without therapeutic intent. The three procedures are informed by previous placebo-control validation work in RCT settings [21]. Procedure (1) is operationalised as the application of a low-velocity broad push manoeuvre to the gluteal region with an inferior-to-superior nontherapeutic line of drive [21]. Procedure (2) consists of a manual manoeuvre involving 3 sets of 10 minimal oscillations (0 to ± 2°) of the flexion-distraction piece with light touch over the lumbar spine region. Procedure (3) involves two-handed bilateral superior-to-inferior scapulae push manoeuvres [21]. Additional file 3C details the placebo-control SMT protocol [23].

### Outcomes

#### Primary outcome

Our primary outcome is participant blinding within each intervention arm immediately after an intervention session, as measured by the Bang blinding index (BI) [13, 24]. The blinding assessment question states ‘As mentioned at the beginning of the study, one of the two study interventions had unknown benefits (genuine treatment) and the other intervention was not known to be beneficial (control treatment). Which treatment do you believe you received?’. There are five response options: ‘Strongly believe I received the genuine treatment’, ‘Somewhat believe I received the genuine treatment’, ‘Somewhat believe I received the control treatment’, ‘Strongly believe I received the control treatment’, and ‘I do not know which treatment I received’. Bang BI estimates (between − 1 to + 1, with 0 suggesting ‘ideal’ blinding—random guess) can be interpreted as the proportion of correct guesses beyond chance within an intervention arm. From arm-specific estimates, a sum Bang BI can be calculated measuring the difference in proportions in the same guess (0 suggesting, for example, an equal proportion of participants believing active treatment in both arms). We deem an absolute arm-specific value score of ≤ 0.3 (i.e. − 0.3 to 0.3) as suggestive of adequate blinding [25].

#### Secondary outcomes

Our first secondary outcome is participant blinding using a second BI metric—the James BI [26]. James BI provides a measure of study-level blinding (i.e., not specific to an intervention arm). The James BI is a modification of the kappa statistic that measures disagreement beyond chance and returns a value between 0 and 1. A value of 1 is returned when all responses are ‘don’t know’ (complete blinding), 0 is returned when all responses are correct (complete unblinding), and 0.5 when responses are randomly distributed (50% correct, 50% incorrect). Secondary outcomes measured after each intervention session include the following: outcome assessor blinding (Bang and James BIs), and factors influencing perceived intervention assignment among participants and outcome assessors (thematic analysis [27], answers to the open-ended questions ‘Why do you believe you received this treatment?’, and ‘Why do you believe this participant received this treatment?’ respectively). Other outcomes are included to blind the study objective from participants and foster impartiality. These are lumbar spine range of motion (ROM, maximum active total flexion and extension), self-rated general health, satisfaction with care, pain intensity, and function. In addition, participant-reported credibility of the interventions and expectancies are measured after the second intervention session with the credibility/expectancy questionnaire (CEQ) [28]. Lastly, to complement the main study objectives and provide exploratory information for the future trial, intervention provider-reported outcomes are also collected after each session. These include intervention component delivery fidelity and quality of intervention delivery relative to the protocol. See Table 1 for the schedule of study procedures related to screening, enrolment, interventions, and assessments.

**Table 1:** Schedule of screening, enrolment, interventions, and assessments

Trial event	Screening	Enrolment	Study visit 1		Study visit 2	
Study visit period			Pre	Post	Pre	Post
Time point—days	− 1	0	0	0	+ 2 to 14	+ 2 to 14
Enrolment						
Eligibility criteria	X					
Patient information and informed expression of interest	X					
Demographics and study entry form	X					
Informed consent in-person signature at clinic		X				
Randomisation		X				
Assessments—intervention providers						
Interventions delivered			X		X	
Intervention component fidelity				X		X
Participant tolerability				X		X
Quality of intervention delivery				X		X
Assessments—participants						
Range of motion			X	X	X	X
General health			X	X	X	X
Pain intensity			X	X	X	X
Back function			X	X	X	X
Satisfaction with care				X		X
Blinding assessment				X		X
Factors influencing beliefs about intervention assignment				X		X
Credibility/expectancy of interventions						X
Assessments—outcome assessors						
Blinding assessment				X		X
Factors influencing beliefs about intervention assignment				X		X

### Sample size consideration

A precision-based approach has been used to decide sample size using the primary outcome measure of the Bang BI estimate (i.e., width of the 95% CI) rather than statistical hypothesis testing/power given the purpose of this blinding feasibility trial [29]. We estimate the Bang BI within each intervention arm and present a 95% CI for each mean BI point estimate. For a sample size of 26 participants per arm, the 95% CI is the observed BI estimate ± 0.225 points (0.45 points width of the 95% CI) for the primary outcome measure of the arm-specific Bang BI, according to Thompson’s method, as described by Landsman and colleagues (see Equation 1 in [30]). Conservatively allowing for attrition of participants of up to 15%, we aim to recruit at least 30 participants per arm (at least 60 participants in total) for this blinding feasibility RCT.

If the minimum recruitment targets for the desired precision are met, and trial participation interest is sufficient to aim for a larger study size, we will consider enrolling and conducting the trial in up to a maximum of 50 participants per arm (100 participants in total). This additional enrolment will yield more precise and informative estimates of the primary outcome.

### Randomisation

#### Allocation sequence generation

A computer-generated randomisation sequence will be generated by an independent statistician at the EBPI and used to randomly allocate participants to active or placebo-control SMT (1:1 ratio). Randomisation will be stratified by SMT experience as a patient or recipient in the past three months, and low back pain in the past four weeks (3 or greater on a 0 out of 10, NRS), and blocked with randomly varying block sizes, which will not be disclosed to ensure concealment of allocation of participants, outcome assessors, data analysts, and investigators.

### Concealment mechanism and implementation

Participants will be allocated to intervention arms through a concealed, central, web-based randomisation system via REDCap; once eligibility is determined, informed consent will be obtained, and baseline data will be entered in an electronic case report form. Intervention providers will assign participants to one of the two SMT interventions—coded ‘A’ and ‘B’—at study visit 1, immediately before delivering the intervention. They will perform randomisation in a private study treatment room with an electronic device and shield the screen from the participants’ view.

### Blinding

Per protocol, participants, outcome assessors, data analysts, and investigators will be blinded to intervention assignment after randomisation. This will be achieved through appropriate trial processes keeping intervention providers and outcome assessors in separate spaces in the study clinic and emphasising the importance of no discussion of study aspects between clinicians, restricting REDCap user right privileges, and by not breaking the code of the intervention variable until two blinded data analyses are completed. Due to the physical nature of both the active and placebo-control SMT interventions, intervention providers will not be able to be blinded to the interventions they are delivering. Yet, they will be trained not to disclose the assigned intervention, nature of the interventions, nor trial objectives to trial participants or other members of the trial team.

### Data collection and instruments

The inclusion and exclusion criteria (eligibility), patient information and informed expression of interest forms, will be completed online, prior to the first study visit. The informed consent form, study entry survey, and pre- and post-intervention assessments will be completed face to face. Trial coordinators will provide participants with a tablet or laptop with the REDCap survey [18] in the study clinic waiting area. Trial coordinators will have completed Good Clinical Practice (GCP) training prior to the start of the trial. The study entry survey includes questions on height, weight, gender, experience with SMT (in the past three months and lifetime), low back pain in the past four weeks, and average pain in the past four weeks. The following outcomes with their corresponding instruments will be captured:

- Range of motion of the lumbar spine: Measured by placing a measuring mobile phone at T12 with the participant standing, using the iOS application *Measure®* (iOS version 16.0.2, iPhone.® model X, Apple Inc., CA, USA) [31–33]
- Self-rated health: Measured with an item from the RAND 36-Item Short-Form Survey Instrument [34]
- Pain intensity: Measured with the NRS [35]
- Back function: Measured with selected items from the International Fitness Scale [36], the Cornell Musculoskeletal Discomfort Questionnaire [37], and a NRS (0 [Worst possible back function] to 10 [Best possible back function])
- Satisfaction with care: Measured by degree of agreement with the statement ‘I am satisfied with the treatment I received today’, with five Likert-type answers—‘Strongly agree’, ‘Agree’, ‘Uncertain’, Disagree’, and ‘Strongly disagree’
- Beliefs about intervention assignment: Measured with blinding questionnaires [13]—a standardised approach for blinding assessment
- Factors influencing beliefs about intervention assignment: Assessed with the open-ended questions ‘Why do you believe you received this treatment?’ (participants) and ‘Why do you believe this participant received this treatment?’ (outcome assessors)
- Credibility/expectancy of interventions: Measured with the CEQ [28]
- Intervention fidelity: Assessed by asking intervention providers to record in case report forms information pertaining to participant tolerance, potential procedure alteration, and occurrence of joint cavitations immediately after intervention delivery
- Quality of intervention delivery: Intervention provider-completed question ‘What number do you feel best describes the quality of the intervention delivery for this participant relative to the intervention protocol you were trained to deliver?’ with 11 possible answers on a 0 to 10 NRS

### Adverse events

All adverse events are appropriately documented and reported following the Clinical Trials Ordinance applicable to the Federal Act on Research involving Human Beings.

### Statistical methods

Blinding analyses will be conducted using an intention-to-treat approach with the R package BI, version 1.1.0 [38, 39]. The current implementation of this package supports both Bang and James BI approaches, the presence of two intervention arms, and three intervention assignment beliefs by participants (i.e., ‘genuine’, ‘control’, and ‘do not know’). Information from the five-level blinding assessment items will be descriptively analysed and tabulated with numbers and percentages. To explore the influence of SMT experience on blinding feasibility of participants and outcome assessors, the responses from the blinding assessment will be described as counts and percentages, by levels of SMT experience. Within- and between-group changes in pain intensity and lumbar ROM will be described, although no formal statistical testing is performed given that these outcomes are collected to blind the study objective from participants and are not relevant to the blinding feasibility assessment. Factors contributing to perceptions about intervention arm assignment among participants and outcome assessors will be analysed using qualitative thematic analysis [27]. The analytical approach is informed by previous feasibility work [40]. Initially, three investigators (blinded to assigned interventions) will independently group similar responses using colour codes. Subsequently, they will develop themes through an iterative and consensus-based process.

To assess the impact of possible deviations from allocated intervention, an as-treated analysis accounting for potential lack of adherence or human errors by intervention providers will be conducted, if applicable and appropriate, to assess blinding outcomes.

## Ethics and dissemination

### Ethical considerations

The study will be conducted in accordance with the protocol and with principles enunciated in the current version of the Declaration of Helsinki, the guidelines of GCP—the international ethical and scientific quality standard for designing, recording, and reporting trials involving human subjects—issued by ICH, the Swiss Law, and Swiss regulatory authority requirements. The independent ethics commission of Canton Zurich (Kantonale Ethikkommission Zürich) approved this trial (BASEC number: 2023–00381). Protocol modifications deemed important will be communicated to the ethics commission and other relevant parties. Individual participant health information collected by this study is considered confidential. Participant confidentiality is ensured by using identification code numbers corresponding to trial and health-related data in the trial database and any output files. Participants will be offered the opportunity to be fully informed of the blinding feasibility objectives and results after the full report is published.

### Patient and public involvement and dissemination

Patients and members of the public were not involved in this blinding feasibility trial protocol due to resource constraints. Yet, patient and clinician perspectives from other preliminary work [41] are helping to inform the developing SALuBRITY trial protocol and indirectly informed this methodological work. Also, patient representatives were involved in developing the recent CoPP statement [7] and found blinding and placebo-controlled trials in the interest of patients. We intend to involve the public in disseminating our results, including via social media platforms, newsletters, conferences, and an open-access publication.

## Discussion

This manuscript presents the rationale and design of a randomised trial investigating the feasibility of blinding participants and outcome assessors to active or placebo-control SMT intervention protocols. This trial also explores the influence of SMT experience and the presence of low back pain on blinding feasibility and describes factors contributing to participants’ and outcome assessors’ perceptions about the interventions assigned.

This blinding feasibility trial protocol has several strengths. First, we prespecify standard operating procedures and rigorous training to safeguard the integrity of our blinding feasibility trial objectives. Second, our blinding feasibility assessment includes outcome assessors that interact with participants and assess their outcomes, but do not deliver the interventions, thereby allowing for the possibility to remain blinded to the interventions assigned after randomisation. Third, we aim to assess blinding at two different timepoints, providing some exploration of blinding in the context of two SMT intervention sessions up to two weeks apart. Assessing blinding at more than one timepoint is relevant as blinding may change over time after several intervention exposures, blinding assessments, and experienced effects [42]. Fourth, the inclusion of a qualitative thematic analysis of factors contributing to perceptions about the assigned intervention will be informative for future RCTs of SMT and other physical interventions.

Overall, the results from this randomised trial assessing the feasibility of blinding SMT interventions among participants and outcome assessors will inform future RCTs in the field of manual medicine. Our findings will directly inform the blinding methods of a future double placebo-controlled trial comparing SMT and corticosteroid nerve root injection for lumbosacral radicular pain—the SALuBRITY trial (ISRCTN87156139).

## Trial status

This protocol is dated April 3, 2023—version 1.3. Recruitment began on April 4, 2023, and was completed on April 25, 2023.

### Supplementary Information

**Additional file 1.** Standard Protocol Items: Recommendations for Interventional Trials (SPIRIT) 2013 Checklist.

**Additional file 2. **Study information and consent form.

**Additional file 3. **Standard operating procedures and intervention descriptions.

## Data Availability

The collected data files and other materials, including full protocol, study forms, participant-level dataset, and statistical code, will be available on reasonable request to the corresponding or first author.

## Abbreviations

BASEC: Business Administration System for Ethics Committees
GCP: Good clinical practice
RCT: Randomised controlled trial
REDCap: Research Electronic Data Capture
ROM: Range of motion
SALuBRITY: Spinal manual therapy versus nerve root injection for lumbosacral radicular pain
SMT: Spinal manual therapy
SPIRIT: Standard Protocol Items: Recommendations for Interventional Trials
TIDieR: Template for Intervention Description and Replication
