# Supplementary material for: Feasibility of blinding spinal manual therapy interventions among participants and outcome assessors: protocol for a blinding feasibility trial
Source: Pilot Feasibility Stud. 2024 May 2;10:70. doi: 10.1186/s40814-024-01492-6 (PMC11064349; doi:10.1186/s40814-024-01492-6)
Supplement: Supplementary file 2 — Additional file 2. Study information and consent form. [file 40814_2024_1492_MOESM2_ESM.pdf]

University of Zurich[UZH]

Balgrist
University Hospital

Additional file 2. Study information and consent form

Study title: **Feasibility of spinal manual therapy interventions for a trial assessing management of primary care patients with back pain: a feasibility randomised controlled trial**

Short title: Feasibility of spinal manual therapy interventions for a future clinical study of back pain

Study organised by: Dr. Cesar Hincapié, DC PhD (Investigator)
University of Zurich and Balgrist University Hospital
Epidemiology, Biostatistics and Prevention Institute and University Spine Centre Zurich
Forchstrasse 340, 8008 Zurich
Phone: +41 44 386 57 29 | Email: cesar.hincapie@uzh.ch

Dear Candidate Participant,

We would like to **inform you about our clinical research study** and **ask if you would like to participate**.

In this study, we want to compare two different manual therapy interventions of the back to find out if they are practical and acceptable for a future study that will investigate the treatment of patients with back pain. The manual therapy interventions we are studying are safe and often used to help with back pain and function in manual medicine healthcare, including chiropractic, physiotherapy, osteopathy, and manual medicine. One of these manual therapy procedures is an active or real treatment, but the exact benefit is not known. The other manual therapy procedure is a comparison or control treatment that is not known to have a benefit.

Your **participation is voluntary**. All information collected in this research study is managed according to strict Swiss data protection laws.

If you decide to participate, please sign the **informed consent form** on page 3. With your signature, you confirm that you have read and understood the study information. If you do not understand something, please ask the study investigator (Dr. Cesar Hincapié) or any of the study contact persons (see bottom of page 2).

Detailed study information to help you decide if you would like to participate:

1. Why are we doing this study?

We want to investigate two manual therapy interventions of the back and their feasibility (practicality and acceptability) for a larger future clinical trial involving patients with back pain. This is a randomised trial. This means that if you participate, you will be randomly assigned to one of two spinal manual therapy interventions.

2. Who can participate in this study?

We will include adults (18 years and older) with or without low back pain, and with or without experience of manual therapy interventions of the back. Participants do not have serious health conditions that prevent them from attending the study clinic or receiving spinal manual therapy interventions, do not have a history of lumbar spine surgery, and are not currently under care or in consultation with a specialist, chiropractor, physiotherapist, or osteopath for back pain. Participants are not currently involved in pending litigation related to back pain or another research study related to backpain. Manual medicine healthcare providers (chiropractors, physiotherapists, massage therapists, osteopaths, or manual medicine physicians), and pregnant or breastfeeding women are not eligible for the study.

3. What is involved if you decide to participate?

The study involves two appointments over 1 to 2 weeks at CHIROMED Praxis im Seefeld (Dufourstrasse 101, 8008 Zurich). Each appointment is about 15 minutes long. As a participant, you will schedule your appointments online at your availability and convenience, between April and May 2023, Monday to Thursday, between 16:00 and 21:00. At each appointment, you will be asked to undress to your underwear and then receive a spinal manual therapy intervention to the low back and mid back, which will take about 4 minutes. You will also be asked a few health-related questions at each appointment.

4. What does a spinal manual therapy intervention involve?

A spinal manual therapy intervention involves a combination of manipulation (a fast controlled movement applied to your low back and mid back) and mobilisation (slower, gentle, massage-like movements). Experienced licensed chiropractors will deliver all spinal manual therapy interventions.

5. Benefits and risks

You may not directly benefit from your participation in this study. However, spinal manual therapy interventions can help with back pain, discomfort, and general function and well-being. The study appointments and interventions will be provided at no cost.

Some people who receive spinal manual therapy experience mild muscle soreness, which typically resolves within 1 to 2 days. Generally, the manual therapy interventions we are studying are safe and often used to help with back pain and function in manual medicine healthcare, including chiropractic, physiotherapy, osteopathy, and manual medicine.

6. Results and after study treatment options

You may choose to receive the results of the study once the trial and scientific report are completed. If during your appointments, a study clinician discovers an issue concerning your health or safety, you will be notified immediately.

If you would like to continue manual therapy treatment after the study, a list of certified chiropractors in Switzerland may be provided or a regular consultation can be arranged in the study clinic Praxis CHIROMED in Seefeld.

7. Confidentiality

Personal and health data will be collected at each appointment and always managed with strict confidentiality. During data collection, your data will be encrypted. Encryption means that all reference data that could identify you, includingyour name and date of birth, are replaced by a code. Only study investigators with access to a secure codebook list candecipher the codes. You have the right to view your data at any time.

This study may be reviewed and audited by the independent Ethics Committee of Canton Zurich (Kantonale Ethikkommission Zürich). If this is the case, your personal and health information may need to be disclosed by law.

8. Rights and withdrawal

You can ask questions about the study at any time. After you have signed the informed consent, you will receive a signed copy by email. Your **participation in this study is voluntary** and you can stop and withdraw from the study at any time, without giving reasons. The data collected up to that point will be analysed and then anonymized, which means that we will delete all information that could identify you. After this process, no one will know that the data came from you.

9. Compensation

You will not receive any compensation for participating in this study. However, you will receive a small token of appreciation (a gift or discount card) at the end of the study, after the second appointment.

10. Liability

If you suffer damage due to participation, the University of Zurich and Balgrist University Hospital will be liable by law.

11. Funding

This study is funded internally at the Epidemiology, Biostatistics and Prevention Institute and the University Spine Centre Zurich. The study does not have external funding.

12. Contact persons

For any questions, concerns, or emergencies related to this study, you can contact one of these four contact persons.

Principal investigator and project leader:
Dr. Cesar Hincapié, DC PhD
University of Zurich and Balgrist University Hospital
Epidemiology, Biostatistics and Prevention Institute
University Spine Centre Zurich
Phone: +41 44 386 57 29
Email: cesar.hincapie@uzh.ch

Study clinic site leader:
Dr. Léonie Hofstetter, DCM
CHIROMED Praxis im Seefeld
Phone: +41 77 420 94 45
Email: leonie.hofstetter@balgrist.ch

Trial coordinator:
Dr. Javier Muñoz Laguna, DC MSc
University of Zurich and Balgrist University Hospital
Epidemiology, Biostatistics and Prevention Institute
University Spine Centre Zurich
Phone: +41 79 616 12 89
Email: javier.munozlaguna@uzh.ch

Trial coordinator:
Astrid Kurmann, MChiroMed
University of Zurich and Balgrist University Hospital
Epidemiology, Biostatistics and Prevention Institute
University Spine Centre Zurich
Phone: +41 79 412 82 16
Email: astrid.kurmann@balgrist.ch

Declaration of consent

Please read this form carefully. Ask the study investigators if there is anything you do not fully understand or if there is anything else you would like to know.

**BASEC number (after submission):**	2023-00381
**Title of the research project:**	Feasibility of spinal manual therapy interventions for a trial assessing management of primary care patients with back pain: a feasibility randomised controlled trial
**Responsible institution:**	University of Zurich and Balgrist University Hospital<br>Epidemiology, Biostatistics and Prevention Institute<br>University Spine Centre Zurich<br>Forchstrasse 340, 8008 Zurich
**Place of implementation:**	CHIROMED Praxis im Seefeld — Dufourstrasse 101, 8008 Zurich
**Head of the research project at the study site:**	Dr. Léonie Hofstetter DCM, Study clinic site leader
**Participant:**<br>Name and first name in block letters:<br>Date of birth (dd.mm.yyyy):	

- The assigned investigator has informed me verbally and in writing about the purpose, the course of the research project, about possible advantages and disadvantages, and about possible risks.
- I am voluntarily participating in this research project and accept the contents of the written information provided on the above research project. I have had enough time to make my decision.
- My questions related to participation in this research project have been answered. I will receive a copy of my electronic informed consent.
- I agree that the responsible project management and the ethics committee in charge of this research project may inspect my unencrypted data for audit and control purposes, but under strict observance of confidentiality.
- I understand that my personal data can only be disclosed in encrypted form. The sponsor guarantees that data protection standards will be maintained according to Swiss legislation.
- I can withdraw from participation at any time and without giving reasons.
- The University of Zurich and Balgrist University Hospital have liability insurance that will cover any damages related to the study.
- I am aware that the obligations stated in the information document must be met. In the interest of my health, the investigator may exclude me at any time.

Place, date	Signature of Participant:

Confirmation by the investigator: I hereby confirm that I have explained the nature, significance, and scope of the research project to this participant. I assure that I will fulfil all obligations in connection with this research project in accordance with the law applicable in Switzerland. If, during the research project, I learn about any aspects that could influence the participants' willingness to participate in the research project, I will immediately inform them.

Place, date	Surname and first name of the investigator in block capitals<br><br>Signature of Investigator:
