# Supplementary material for: Feasibility of blinding spinal manual therapy interventions among participants and outcome assessors: protocol for a blinding feasibility trial
Source: Pilot Feasibility Stud. 2024 May 2;10:70. doi: 10.1186/s40814-024-01492-6 (PMC11064349; doi:10.1186/s40814-024-01492-6)
Supplement: Supplementary file 3 — Additional file 3. Standard operating procedures and intervention descriptions. [file 40814_2024_1492_MOESM3_ESM.pdf]

Additional file 3. Standard operating procedures and intervention descriptions

Table of contents

A. Standard Operating Procedures 2
B. Intervention Description (TIDieR checklist) – Active SMT 6
C. Intervention Description (TIDieR and TIDieR-Placebo checklists) – Placebo-control SMT 8

A. Standard Operating Procedures

Time	Process	Description
T_{-4}	Recruitment – invitation and interest	**Study team member introduces study to candidate participant and asks for interest.**<br><br>Inclusion criteria:<br>• men and women, aged 18 years or older<br>• with or without current low back pain<br>• with or without experience of spinal manual therapy (SMT)<br><br>Exclusion criteria:<br>• pregnant or breastfeeding<br>• manual medicine healthcare provider (i.e., chiropractor, physiotherapist, osteopath, massage therapist, manual medicine physician)<br>• history of lumbar spine surgery or serious spinal pathology<br>• serious comorbidity preventing study participation<br>• currently under care or in consultation with a specialist, chiropractor, physiotherapist, or osteopath for back pain<br>• currently participating in another research study related to back pain<br>• currently involved in pending litigation related to back pain<br><br>What's involved?<br>• 2 appointments over 1 to 2 weeks at CHIROMED Praxis im Seefeld (Dufourstrasse 101, 8008 Zurich), each 15 to 20 minutes long (at least 1 day in between appointments, and maximum of 2 weeks)<br>• appointments are self-scheduled by participant online at their convenience, from 27 March 2023 to 20 April 2023, Monday to Thursday, between 16:00 and 21:00<br>• at each appointment, participant will receive a spinal manual therapy intervention to the low back and mid back, which will take about 4 minutes<br>• participant is asked a set of health-related questions at each appointment, before and after the intervention<br><br>Key points:<br>• candidates must express interest to consider participation<br>• request active email address to send study information form and link to schedule appointments<br>• candidate name and contact email address entered in study candidates list

Time	Process	Description
T_{-3}	Recruitment – email invitation	**Study team member sends study invitation email to candidate for fully informed consideration and survey link to book two appointments at the clinic.**<br><br>Study invitation email is a **standardised message highlighting main eligibility criteria** with **study information and consent form** attachment (both DE and EN versions).<br>Study invitation email provides a **Doodle link** for candidate participants to schedule two study appointments at their availability and convenience at the study clinic.
T_{-2}	Recruitment – eligibility, e-consent and enrollment	**Candidates with scheduled appointments receive a REDCap survey link to self-assess eligibility and request electronic consent (e-consent) if interested and willing to participate.**<br><br>In REDCap system, candidates complete a survey to self-assess eligibility.<br>If eligibility criteria are met, candidates are directed to the electronic consent framework in REDCap, containing the study information form (already familiar to candidates from step T_{-3}), a question explicitly asking for consent, their first name, family name, email, date of birth, electronic signature, place, and date of consent.<br>Once participants complete their e-consent, they are officially enrolled in the trial and receive a copy of their signed consent via email.
T_{-1}	Pre-visit 1 – email	**Participant receives a standardised email requesting completion of study entry form and reminder of their scheduled clinic appointments.**<br><br>Email provides REDCap link to study entry form and full details for booked appointments.
T_0	Visit 1 – Admin and baseline survey	**Participant is welcomed at the clinic, receives a Study ID sticker (participant number), and completes pre-intervention questions.**<br><br>Participant arrives at the clinic and is welcomed by the trial coordinator, who provides the Study ID sticker: 'Please place this Study ID sticker over your left chest area.'<br>Managing clinician checks that the Study Entry Form was filled out correctly by the participant and completes a 'Data quality check' to confirm that the two questions informing the stratification variables for randomisation have been answered by the participant.<br>Trial coordinator introduces participant to managing clinician, who then guides participant to the clinic area where participant completes the Pre-intervention questions.<br>Once done, participant is directed to the treatment room by the managing clinician.

Time	Process	Description
$T_{0.1}$	Visit 1 – Pre-intervention range of motion assessment	**Participant and managing clinician enter the treatment room for range of motion assessment.**<br><br>In the treatment room, the managing clinician asks the participant to undress, leaving their underwear on. Once the participant is undressed, the managing clinician conducts the back range of motion assessment.<br>Managing clinician asks the participant to keep their feet together and adopt an upright position, and then places the measuring device (iPhone) at the level of T12, after palpating the lowermost ribs.<br>Managing clinician instructs the participant to achieve maximum active flexion range of motion: 'Keeping your knees straight, please bend forward as far as you can go.' Then, returns to neutral upright position after maximum flexion: 'Please return to standing upright and neutral.'<br>Managing clinician records the degrees of flexion without disclosing the value to the participant.<br>Managing clinician instructs the participant to achieve maximum active extension range of motion: 'Keeping your knees straight, bend back as far as you can go.' Then, returns to neutral upright position after maximum extension: 'Please return to standing upright and neutral.'<br>Managing clinician records the degrees of extension without disclosing the value to the participant.<br>Managing clinician instructs the participant of next phase and leaves the room: 'The treating clinician will be with you shortly. Please have a seat on the treatment table while waiting.'<br>At this stage, the trial coordinator directs the managing clinician to the managing clinician room (an assigned area of the clinic, separate from the treatment room and the treating clinician room).
$T_{0.2}$	Visit 1 – Intervention session 1	**Treating clinician enters the treatment room, performs randomization, and delivers the intervention.**<br><br>Under coordination by the trial coordinator, the treating clinician enters the treatment room: 'Hello, my name is Dr. ___________, I will be your treating clinician today. We will now go ahead and perform your spinal manual therapy treatment.'<br>Treating clinician performs randomisation: 'Please give me a second to check some information here [performs randomisation on a relevant device away from the participant's view; randomisation output is either 'A' or 'B', and only the treating clinician knows the allocated intervention].'<br>Treating clinician delivers either active or placebo-control spinal manual therapy intervention, as specified in the TIDieR descriptions included in the **Appendix**.<br>Verbal exchanges between treating clinician and participant are pragmatic, neutral, and non-revealing of the assigned intervention for adequate intervention delivery.<br>Example commands include: 'Please lie on your side facing me', 'Please roll over and lie on your other side', 'Please now lie face down'.<br>Once intervention delivery is completed, the treating clinician instructs the participant of next phase and leaves the room: 'We are all done, you can now sit up. The managing clinician will back with you in a moment.'<br>At this stage, the trial coordinator directs the treating clinician to the treating clinician room (an assigned area of the clinic, separate from the treatment room and managing clinician room).
$T_{0.3}$	Visit 1 – Post-intervention range of motion assessment and managing clinician questions	**Managing clinician re-enters the treatment room for post-intervention range of motion assessment.**<br><br>Under coordination by the trial coordinator, the managing clinician re-enters the treatment room. During this important transition, the trial coordinator ensures that there is no exchange whatsoever between the treating and managing clinicians.<br>Once the managing clinician is in the room, the post-intervention range of motion assessment is performed following the same sequence as in $T_{0.1}$.<br>Once completed, the managing clinician instructs the participant to dress: 'You can now get dressed again. Once ready, please see the trial coordinator outside the treatment room for a final short survey that needs to be completed.'<br>Managing clinician leaves the treatment room and returns to the managing clinician room to complete the managing clinician survey on REDCap.

Time	Process	Description
$T_{0.4}$	Visit 1 – Post-intervention survey and admin	**Participant completes a post-intervention survey, is thanked and provided guiding information for next study visit.**<br><br>Participant completes the 'Post-intervention questions' survey on REDCap.<br>Participant is guided to the exit of the clinic by the managing clinician and trial coordinator.<br>Participant is thanked for their participation and reminded of their next appointment details.<br>Participants are offered a small token of appreciation after study visit 1 (chocolate or similar).
$T_{0.5}$	Pre-visit 2 – reminder email	**Participant receives pre-visit email reminder before scheduled appointment for study visit 2.**<br><br>Participant receives email reminder with details of their second study appointment.
T_1	Visit 2 – Admin and baseline survey	**Participant is welcomed at the clinic and completes pre-intervention questions – study visit 2.**<br><br>This step occurs as described in T_0.
$T_{1.1}$	Visit 2 – Pre-intervention range of motion assessment	**Participant and managing clinician enter the treatment room for range of motion assessment.**<br><br>This step occurs as described in $T_{0.1}$.
$T_{1.2}$	Visit 2 – Intervention session 1	**Treating clinician enters the treatment room and delivers the intervention – study visit 2.**<br><br>This step occurs similar to $T_{0.2}$ except without randomisation procedures. Important that the treating is well aware of the randomly assigned intervention to be delivered at study visit 2 (same as visit 1).
$T_{1.3}$	Visit 2 – Post-intervention range of motion assessment	**Managing clinician re-enters the treatment room for post-intervention range of motion assessment.**<br><br>This step occurs as described in $T_{0.3}$.
$T_{1.4}$	Visit 2 – Post-intervention survey and admin	**Participant completes post-intervention questions for study visit 2.**<br><br>Participants complete the post-intervention survey for study visit 2 (similar to $T_{0.4}$ procedure).
T_2	Debriefing and trial closure	**Participant is offered the opportunity to be fully informed of the blinding feasibility trial objectives. Participant is thanked for their participation and their trial is closed.**<br><br>Participants is offered more information about the primary trial objectives and asked if they would like to receive the final trial report via email.<br>Participant receives a small token of appreciation (Decathlon gift card and small backpack) for participating in the study.

B. Intervention Description (TIDieR checklist)[1] – Active SMT

1. **Brief name:** Provide the name or a phrase that describes the intervention
   Active spinal manual therapy (SMT).

2. **Why:** Describe any rationale, theory, or goal of the elements essential to the intervention
   Active SMT is a pragmatic therapeutic concept combining active lumbar spine mobilisation and manipulation, and thoracic spine manipulation, tailored to participant intervention comfort and tolerance. Active SMT imparts direct and indirect effects aimed at improving musculoskeletal pain and function, through multiple proposed neurophysiological mechanisms. A key essential element of the manipulation component of active SMT entails an intentional high-velocity, low-amplitude (HVLA) force applied to a target joint segment. For the mobilisation component, an essential element involves purposeful application of low-velocity, low-amplitude forces to a target joint segment, with sufficient flexion and distraction to induce therapeutic effects. The intervention is delivered in a real-world clinical setting by a treating clinician — a chiropractor not blinded to the allocated treatment who maintains a neutral tone and follows a standard script for interactions with study participants.

3. **Materials:** Describe any physical or informational materials used in the intervention, including those provided to participants or used in intervention delivery or in training of intervention providers. Provide information on where the materials can be accessed (such as URL)
   No informational materials will be used in the active intervention for the participants. Standard chiropractic tables with adjustable height and manual flexion-distraction pieces (ErgoStyle™ FX Flexion) will be used for intervention delivery. One conventional chiropractic table per treating clinician and private practice treatment room will be used for the purpose of this feasibility trial.
   This active intervention description form is used for clinician training and provided to treating clinicians in both electronic and hardcopy formats. Online videos of the intervention protocols are also provided to treating clinicians (video link in item 4 below).

4. **Procedures:** Describe each of the procedures, activities, and processes used in the intervention, including any enabling or support activities
   Active SMT will involve three ordered procedures: (1) active side-posture manipulation of the lumbar spine, (2) active prone lumbar mobilization, and (3) active prone thoracic manipulation. During (1) active side-posture manipulation of the lumbar spine, the clinician will place their contact hand on a lower level of the lumbar region (L4-L5 or L5-S1) and then apply a fast, controlled HVLA thrust with a therapeutic line of drive to the joint segment. Side-lying lumbar manipulation will be performed bilaterally, with the treating clinician choosing any suitable technique, and with or without occurrence of the characteristic audible joint cavitation associated with spine manipulation. (2) Active prone lumbar mobilization will involve three sets of five repetitions of flexion-distraction moves over the same segments (L4-L5 or L5-S1), with a short break (5 to 10 seconds) between the sets. Lastly, (3) active prone thoracic manipulation will involve a HVLA maneuver to the mid-thoracic spine (T5-T6 or T6-T7). Although active SMT is usually administered as part of multimodal care, adjacent to other co-interventions such as education, advice, or exercise prescription, for the purposes of this feasibility trial, active SMT will be restricted to the three procedures described above.

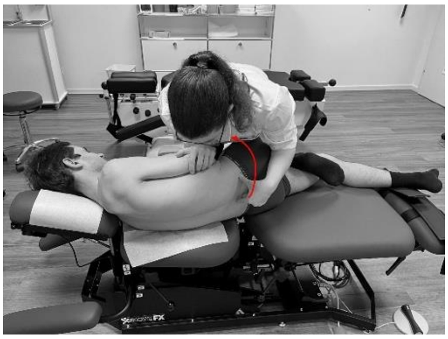
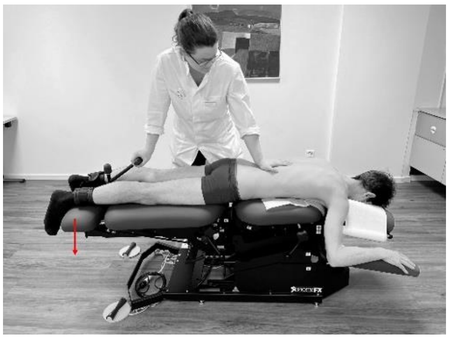
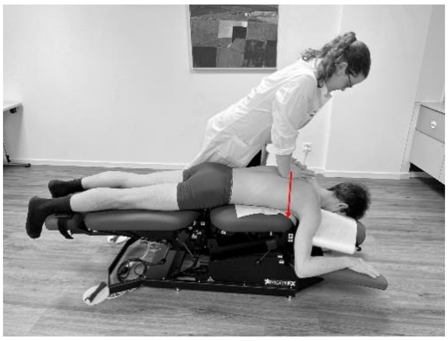

5. **Who provided:** For each category of intervention provider describe their expertise, background, and any specific training given
   Intervention providers (treating clinicians) will be up to 10 licensed chiropractors, with a minimum of 3 years of active clinical experience after full licensure in Switzerland. Chiropractors trained in Switzerland are required to have a Bachelor of Medicine degree, a Master of Chiropractic Medicine degree, and must have completed an assistantship (residency) program. Alternatively, chiropractors may obtain training abroad and require additional requirements upon arrival to Switzerland to

obtain a license to practice. All treating clinicians involved in the trial are trained to standardize the delivery and implementation of the trial interventions.

6. **How:** Describe the modes of delivery (such as face to face or by some other mechanism, such as internet or telephone) of the intervention and whether it was provided individually or in a group
The mode of delivery will be face to face and the intervention will be provided individually and privately through a clinician-participant encounter.

7. **Where:** Describe the type(s) of location(s) where the intervention occurred, including any necessary infrastructure or relevant features
The interventions will occur at a single SALuBRITY trial chiropractic research clinic (private practice) in the canton of Zurich – CHIROMED Praxis im Seefeld (Dufourstrasse 101, 8008 Zurich). The clinic has sufficient private room spaces to meet trial infrastructure and implementation needs. At a minimum, this clinic has the infrastructure to support one to three treating clinicians providing the interventions concurrently, one to three managing clinicians, and one to three trial coordinators to guide participants during the study visits.

8. **When and how much:** Describe the number of times the intervention was delivered and over what period of time including the number of sessions, their schedule, and their duration, intensity, or dose
Two active SMT intervention sessions will be delivered over a four-week period between 27 March 2023 and 20 April 2023. A minimum of one day and a maximum of two weeks may be left between the two scheduled intervention sessions. No specific instructions will be provided to participants between the two sessions. Each study participant will take 15 minutes to complete all study processes (i.e., pre-intervention survey and processing, trial intervention, post-intervention survey and processing) at the trial research clinic for each of the study visits, with the active SMT intervention phase of the study visit lasting approximately 4 minutes in total.

9. **Tailoring:** If the intervention was planned to be personalized or adapted, then describe what, why, when, and how
The delivery will follow a well-structured ordered sequence of three manual procedures (see item 4). Although adherence to the ordered sequence of procedures described in item 4 is desired, as a pragmatic therapeutic concept, active SMT may be adapted and individually tailored, if it does not compromise the main elements of the intervention. For example, in symptomatic participants, active side-posture manipulation of the lumbar spine may be avoided in cases where pain or other existing symptoms may be exacerbated by body positioning during the intervention delivery. Generally, active side-posture manipulation of the lumbar spine, active prone lumbar mobilization, and active prone thoracic mobilization may be customized to the tolerance and comfort of the study participants.

10. **Modifications:** If the intervention was modified during the study, describe changes (what, why, when, and how)
During the study period, no modifications to the active SMT intervention are expected, other than the allowed tailoring described in item 9.

11. **How well:** Planned: If intervention adherence or fidelity was assessed, describe how and by whom, and if any strategies were used to maintain or improve fidelity, describe them
The trial coordinator in the clinic will oversee trial activities, primarily with respect to time dedicated to each process. The one-hour training for SALuBRITY Blinding clinicians is a strategy designed to maintain and improve fidelity to the active SMT intervention. Nevertheless, for each individual active SMT session, the treating clinician will record intervention adherence and fidelity. In cases where the standardized active SMT session needs to be modified to participant's tolerability, this will also be recorded electronically by the clinician providing the intervention. Each experienced treating clinician will also be assigned a unique identifier in the electronic system.

12. **How well:** Actual: If intervention adherence or fidelity was assessed, describe the extent to which the intervention was delivered as planned
To be completed after trial conduct and implementation, on the basis of intervention adherence and fidelity information to be gathered during the trial.

Reference:

1. Hoffmann TC, Glasziou PP, Boutron I, Milne R, Perera R, Moher D, et al. Better reporting of interventions: template for intervention description and replication (TIDieR) checklist and guide. BMJ. 2014 Mar 7;348:g1687.

C. Intervention Description (TIDieR and TIDieR-Placebo checklists)[1,2] – Placebo-control SMT

1. **Brief name:** Provide the name or a phrase that describes the placebo/sham intervention.
   Placebo-control spinal manual therapy (SMT).

2. **Why:** Describe any rationale, theory, or goal of the elements essential to the placebo/sham intervention.
   Placebo-control SMT is conceptualized as the non-therapeutic application of manual maneuvers mimicking active SMT, delivered without therapeutic intent. This is operationalized as the use of low-velocity, low-amplitude maneuvers, and low-grade prone mobilization. Placebo-control SMT shares some elements common to active SMT, including the clinician delivering the intervention, the range of motion assessment, the anatomical regions treated, and the duration of the sessions. Although inertness of the placebo-control SMT intervention cannot be guaranteed, its proposed design and intent is essentially non-therapeutical. The placebo-control intervention is delivered in a real-world clinical setting by a treating clinician — a chiropractor not blinded to the allocated treatment who maintains a neutral tone and follows a standard script for interactions with study participants.

3. **Materials:** Describe any physical or informational materials used in the placebo/sham intervention, including those provided to participants or used in intervention delivery or in training of intervention providers. Provide information on where the materials can be accessed (such as an online appendix, URL).
   No informational materials will be used in the placebo-control intervention for the participants. Standard chiropractic tables with adjustable height and manual flexion-distraction pieces (ErgoStyle™ FX Flexion) will be used for intervention delivery. One conventional chiropractic table per treating clinician and private practice treatment room will be used for the purpose of this feasibility trial.
   This placebo-control intervention description form is used for clinician training and provided to treating clinicians in both electronic and hardcopy formats. Online videos of the intervention protocols are also provided to treating clinicians (video link in item 4 below).

4. **Procedures:** Describe each of the procedures, activities, and/or processes used in the placebo/sham intervention, including any enabling or support activities.
   Placebo-control SMT will involve three ordered procedures: (1) placebo-control side-posture manipulation of the lumbar spine, (2) placebo-control prone lumbar mobilization, and (3) placebo-control prone thoracic manipulation. During (1) placebo-control side-posture manipulation of the lumbar spine, the clinician will place their contact hand broadly on the gluteal region and then apply a low-velocity, low-amplitude push maneuver following a non-therapeutic inferior to superior line of drive to the soft tissue. The lumbar spine will be kept more neutral than in active SMT. The same maneuver will be repeated on the contralateral side, and joint cavitations are not expected. (2) Placebo-control prone lumbar mobilization will involve broad light touch to the lumbar area and three sets of ten low-grade repetitions of flexion-distraction moves – maximum of 5 degrees of flexion and no distraction. A short break (5 to 10 seconds) will be included between sets. Lastly, (3) placebo-control prone thoracic manipulation will involve two low-velocity low-amplitude, and low-force push maneuvers to the scapulae, following a strict superior to inferior line of drive. Placevo-control SMT will be restricted to the three procedures described above.

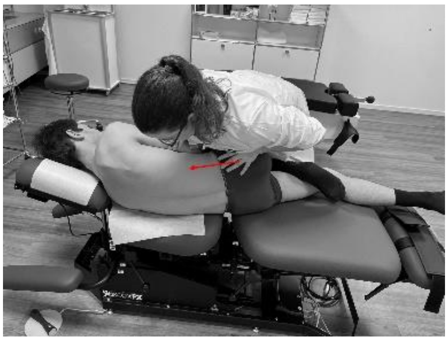
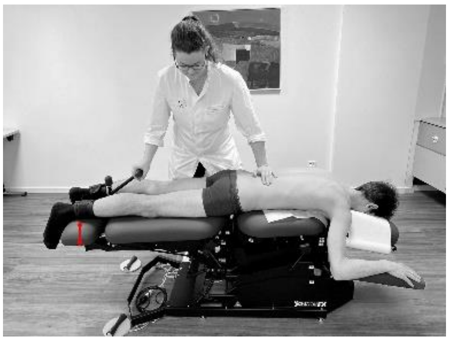
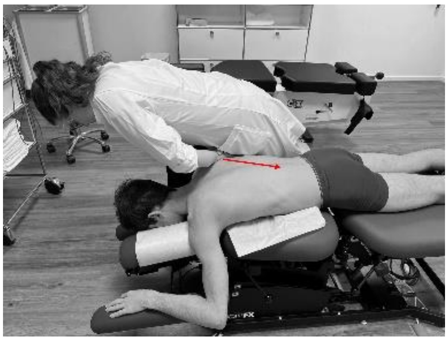

5. **Who provided:** For each category of placebo/sham intervention provider (such as psychologist, nursing assistant), describe their expertise, background, and any specific training given.
   Intervention providers (treating clinicians) will be up to 10 licensed chiropractors, with a minimum of 3 years of active clinical experience after full licensure in Switzerland. Chiropractors trained in Switzerland are required to have a Bachelor of Medicine degree, a Master of Chiropractic degree, and must have completed an assistantship (residency) program. Alternatively, chiropractors may obtain training abroad and require additional requirements upon arrival to Switzerland to obtain a license to practice. All treating clinicians involved in the trial are trained to standardize the delivery and implementation of the trial interventions.

6. How: Describe the modes of delivery (such as face to face or by some other mechanism, such as internet or telephone) of the placebo/sham intervention and whether it was provided individually or in a group.
The mode of delivery will be face to face and the intervention will be provided individually and privately through a clinician-participant encounter.

7. Where: Describe the type(s) of locations(s) and settings where the placebo/sham intervention occurred, including any necessary infrastructure or relevant features.
The interventions will occur at a single SALuBRITY trial chiropractic research clinic (private practice) in the canton of Zurich – CHIROMED Praxis im Seefeld (Dufourstrasse 101, 8008 Zurich). The clinic has sufficient private room spaces to meet trial infrastructure and implementation needs. At a minimum, this clinic has the infrastructure to support one to three treating clinicians providing the interventions concurrently, one to three managing clinicians, and one to three trial coordinators to guide participants during the study visits.

8. When and how much: Describe the number of times the placebo/sham intervention was delivered and over what period of time including the number of sessions, their schedule, and their duration, intensity, or dose. If relevant, include the duration of the pre-, and post-randomisation consultations.
Two placebo-control SMT intervention sessions will be delivered over a four-week period between 27 March 2023 and 20 April 2023. A minimum of one day and a maximum of two weeks may be left between the two scheduled intervention sessions. No specific instructions will be provided to participants between the two sessions. Each study participant will take 15 minutes to complete all study processes (i.e., pre-intervention survey and processing, trial intervention, post-intervention survey and processing) at the trial research clinic for each of the study visits, with the placebo-control SMT intervention phase of the study visit lasting approximately 4 minutes in total.

9. Tailoring: If the placebo/sham intervention was planned to be personalised, titrated or adapted, then describe what, why, when, and how.
The delivery will follow a well-structured ordered sequence of three manual procedures (see item 4). Although adherence to the ordered sequence of procedures described in item 4 is desired, as a pragmatic therapeutic concept, active SMT may be adapted and individually tailored, if it does not compromise the main elements of the intervention. For example, in symptomatic participants, placebo-control side-posture manipulation of the lumbar spine may be avoided in cases where pain or other existing symptoms may be exacerbated by body positioning during the intervention delivery. Generally, placebo-control side-posture manipulation of the lumbar spine, placebo-control prone lumbar mobilization, and placebo-control prone thoracic mobilization may be customized to the tolerance and comfort of the study participants.

10. Modifications: If the placebo/sham intervention was modified during the course of the study, describe the changes (what, why, when, and how).
During the study period, no modifications to the active SMT intervention are expected, other than the allowed tailoring described in item 9.

11. How well: Planned: If placebo/sham intervention adherence or fidelity was assessed, describe how and by whom, and if any strategies were used to maintain or improve fidelity, describe them.
The trial coordinator in the clinic will oversee trial activities, primarily with respect to time dedicated to each process. The one-hour training for SALuBRITY Blinding clinicians is a strategy designed to maintain and improve fidelity to the placebo-control SMT intervention. Nevertheless, for each individual placebo-control SMT session, the treating clinician will record intervention adherence and fidelity. Accidental cavitations during placebo-control SMT will also be recorded in REDcap. In cases where the standardized placebo-control SMT session needs to be modified to participant's tolerability, this will also be recorded electronically by the clinician providing the intervention. Each experienced treating clinician will also be assigned a unique identifier in the electronic system.

12. How well: Actual: If placebo/sham intervention adherence or fidelity was assessed, describe the extent to which the intervention was delivered as planned.
To be completed after trial conduct and implementation, on the basis of intervention adherence and fidelity information to be gathered during the trial.

13. Measuring the success of blinding: Was blinding measured, and if so: how, and what were the results of such measurement?
By protocol, blinding of participants and managing clinicians will be measured following Bang and James approaches.[3,4] The results of such measurements will be reported and included in this form after trial conduct and implementation.

References:

1. Hoffmann TC, Glasziou PP, Boutron I, Milne R, Perera R, Moher D, et al. Better reporting of interventions: template for intervention description and replication (TIDieR) checklist and guide. BMJ. 2014 Mar 7;348:g1687.
2. Howick J, Webster RK, Rees JL, Turner R, Macdonald H, Price A, et al. TIDieR-Placebo: A guide and checklist for reporting placebo and sham controls. PLOS Med. 2020 Sep 21;17(9):e1003294.
3. Bang H, Flaherty SP, Kolahi J, Park J. Blinding assessment in clinical trials: a review of statistical methods and a proposal of blinding assessment protocol. Clin Res Regul Aff. 2010 Jun 1;27(2):42–51.
4. James KE, Bloch DA, Lee KK, Kraemer HC, Fuller RK. An index for assessing blindness in a multi-centre clinical trial: disulfiram for alcohol cessation--a VA cooperative study. Stat Med. 1996 Jul 15;15(13):1421–34.
